# *Allium cepa* L. Inoculation with a Consortium of Plant Growth-Promoting Bacteria: Effects on Plants, Soil, and the Autochthonous Microbial Community

**DOI:** 10.3390/microorganisms9030639

**Published:** 2021-03-19

**Authors:** Marika Pellegrini, Daniela M. Spera, Claudia Ercole, Maddalena Del Gallo

**Affiliations:** 1 AGIRE Soc. Cons. a r.l., Via Isidoro e Lepido Facii, 64100 Teramo, Italy; danielamspera@gmail.com; 2 Department of Life, Health and Environmental Sciences, University of L’Aquila, Via Vetoio, Coppito, 67010 L’Aquila, Italy; claudia.ercole@univaq.it (C.E.); maddalena.delgallo@univaq.it (M.D.G.)

**Keywords:** biostimulants, PGPB, seed inoculation, sustainable agriculture, illumina sequencing

## Abstract

The present work was aimed at investigating the effects of a four bacterial strain consortium—*Azospirillum brasilense, Gluconacetobacter diazotrophicus, Herbaspirillum seropedicae*, and *Burkholderia ambifaria*—on *Allium cepa* L. and on soil health. The bacterial consortium was inoculated on seeds of two different onion varieties; inoculated and Control seeds (treated with autoclaved inoculum) were sown in an open-field and followed until harvest. Plant growth development parameters, as well as soil physico–chemical and molecular profiles (DNA extraction and 16S community sequencing on the Mi-Seq Illumina platform), were investigated. The results showed a positive influence of bacterial application on plant growth, with increased plant height (+18%), total chlorophylls (+42%), crop yields (+13%), and bulb dry matter (+3%) with respect to the Control. The differences between Control and treatments were also underlined in the bulb extracts in terms of total phenolic contents (+25%) and antioxidant activities (+20%). Soil fertility and microbial community structure and diversity were also positively affected by the bacterial inoculum. At harvest, the soil with the presence of the bacterial consortium showed an increase in total organic carbon, organic matter, and available phosphorus, as well as higher concentrations of nutrients than the Control. The ecological indexes calculated from the molecular profiles showed that community diversity was positively affected by the bacterial treatment. The present work showed the effective use of plant growth-promoting bacteria as a valid fertilization strategy to improve yield in productive landscapes whilst safeguarding soil biodiversity.

## 1. Introduction

One of the current concerns in agriculture is to improve the sustainability of productive land and at the same time to achieve high production rates. To achieve this goal, one of the promising and sustainable innovations could be the use of natural plant biostimulants [1]. Plant biostimulants are substances or microorganisms applied to plants for the enhancement of nutrition, tolerance to abiotic stress, and improvement in crop quality traits [2]. Plant biostimulants can be classified into nine categories based on their non-microbial and microbial natures and origin [3]. Among them, plant growth-promoting bacteria (PGPB) are considered sustainable biostimulant agents, effective also in the presence of abiotic stress factors [1]. Several strains are associated with the PGPB group based on their plant growth-promoting (PGP) traits. Among them, key traits are nitrogen fixation, nutrient-solubilising capabilities, and the production of phytohormones [4,5]. PGPB can be applied on seeds or to soil through several formulation (e.g., encapsulated, inorganic, liquid, organic, polymeric), which can influence the biostimulant effectiveness [6]. However, seed inoculation is the most common method of application [6]. In this work, we investigated the biostimulating efficacy of seed inoculation with a bacterial consortium consisting of *Azospirillum brasilense* Cd, *Burkholderia ambifaria* PHP7, *Gluconacetobacter diazotrophicus* Pal5, and *Herbaspirillum seropedicae* Z67. *A. brasilense* belongs to one of the most studied PGPB genera [7] with good biofertilisation potential [8,9,10,11,12,13,14]. The mechanisms of action by which *Azospirillum* spp. improve plant growth involve the production of phytohormones, N_2_-fixation, the mobilization of minerals, the production of small molecules and enzymes, the proliferation of the root system, the improvement of membrane activity, and the uptake of water and minerals [7]. To *Gluconacetobacter* spp. are attributed N_2_-fixation, the production of siderophores and phytohormones, and the solubilization of inorganic forms of phosphorus and zinc [15]. In addition to phytohormones and siderophore production and N_2_-fixation, *Herbaspirillum* spp. and PGP *Burkholderia* species help plants to thrive thanks to the presence of 1-aminocyclopropane-1-carboxylate (ACC) deaminase, which reduces stressful ethylene levels [16,17,18]. In addition, direct and indirect biocontrol capabilities of bacterial and fungal phytopathogens have been reported for all these strains [7,15,16,18]. The production of bacterial consortia, with the combination of two or more strains with PGP characteristics, can increase the effectiveness of the inoculum [19,20]. The combination of *A. brasilense*, *B. ambifaria*, *G. diazotrophicus,* and *H. seropedicae* strains has already shown good biostimulation effects on *Lycopersicon esculentum* L. [21] *Cannabis sativa* L. [22] *Artemisia eriantha* Ten [23], and ancient *Triticum* genotypes [24]. In the present work, we hypothesised that this PGPB consortium could positively affect the growth, development, yield, and quality traits of *Allium cepa* L. crops. To demonstrate the validity of this hypothesis, two onion cultivars suitable for the experimental area were chosen, “Meranto” and “Moondance”. First, bacterial association on inoculated seeds and colonization of seedlings grown in vitro were evaluated by Scanning Electron Microscope (SEM). Then, two parallel one-year field experiments were carried out to evaluate the efficacy of the PGPB treatment on plants (i.e., chlorophyll, length, dry weight) and bulbs (i.e., dry weight, total phenolic content, antioxidant activity) of both onion varieties. To evaluate the influence of this treatment on soil microbial communities, soil samples were also analyzed in terms of physico–chemical and molecular profiles.

## 2. Materials and Methods

### 2.1. Bacterial Strains and Growth Conditions

Meranto (Bejo Italia Srl, Ravenna, Italy) and Moondance F1 (Vilmorin, Paris, France) onion cultivars were utilized in the present study. Bacteria were grown in T4 medium [21] with constant shaking at 30 °C for 24 h. The cell density of each strain was determined spectrophotometrically, by comparing optical densities at 600 nm with calibration curves. The four strains were mixed to obtain an equal number of cells for each strain and a working inoculum with a final density of 10^10^ cells mL^−1^. Seeds were treated with the inoculum by immersion for 20 min. Then, the seeds were removed from the inoculum and dried overnight at 30 °C. The final bacterial density on seeds was 10^6^ cells g^−1^ (determined by a plate count on T4). The Control consisted of seeds inoculated with the same procedure, but with autoclaved inoculum.

### 2.2. Bacterial Adhesion on Seed Surfaces Investigated by Scanning Electron Microscopy

Seeds were sterilized following the procedure previously described [24]. Sterilized seeds were inoculated with the 10^10^ mL^−1^ bacterial suspension for 20 min and allowed us to dry overnight at 30 °C. Control seeds were treated with the same procedures but with a bacterial suspension autoclaved for 15 min at 120 °C. Surfaces and sections (obtained with a sterile lancet) of Control and inoculated seeds were analyzed the day of inoculation and 30 days later. Samples were directly positioned on metal sample holders by double-sided adhesive carbon tape in thermal contact with a Peltier cooling stage maintained at −1 °C. Samples were then analyzed by a Gemini500 scanning electron microscope (SEM—Zeiss, Oberkochen, Germany) operating in wet mode conditions (working distance 6.5–8 mm, variable pressure, accelerating voltage 10 kV, relative humidity 80%, back-scatter electron detector).

### 2.3. Experimental Design and Experimental Conditions

Two parallel field experiments were carried out during the growing season, February–August 2019, at the Agricola Scipioni fields (Avezzano, Italy, 650 m above sea level), characterised by a relatively continental climate, with an average annual rainfall range of 650–800 mm. The onion seeds were sown in February 2019 with a pneumatic seeder (Serie SNT, Agricola Italiana SNC, Massanzago, PD, Italy), 250 seeds m^2^, with five rows of tandem plants spaced 5 cm apart. The previous crop was potato (*Solanum tuberosum* L.), harvested in mid-October 2018. The fields were previously fallow, plowed, and harrowed twice before sowing. Two experimental conditions were compared: seeds treated with the bacterial consortium (PGPB) and seeds treated with the autoclaved consortium (Control). The experimental design consisted of a split plot design with five replications, where onion cultivars (Meranto and Moondance) represented the main plots and seed treatment (PGPB and Control) the sub-plots. Each experimental unit covered an area of 5.0 m × 6.0 m (30 m^2^).

### 2.4. Crop Monitoring and Sampling

From 15 days after sowing (DAS) up to 150 DAS, plant height was monitored and aerial part samples were collected randomly within each experimental unit. The aerial parts were taken to the laboratory and analyzed for dry weight (80 °C to constant weight) and chlorophyll content [25]. At harvest (180 DAS), bulbs were collected and yields (t ha^−1^) estimated. Bulb samples were randomly collected within each experimental unit, taken to the laboratory, and processed for further investigations as described in Section 2.5.

### 2.5. Bulb Analyses

Bulbs were investigated for their dry weight, total phenolic contents, and antioxidant activity. Dry weight was obtained by heat treatment of bulb slices at 80 °C to constant weight. For total phenolic content and antioxidant activity, bulb slices were immediately frozen at −80 °C and freeze-dried completely (15 h). Lyophilized samples were then hermetically packed in high-barrier plastic bags and kept at −80 °C until analysis. Five replicates per each sample were extracted by 1 min microwave extraction (350 W) with a 50:50 (*v/v*) ethanol–water solution and a sample–solution ratio of 1:25. The total phenolic content assay was carried out utilizing Folin–Ciocâlteu reagent and the Singleton and Rossi procedure [26]. Gallic acid was used as a reference standard, and results were expressed as mg GAE (gallic acid equivalents) g^−1^ DW (dry weight). Antioxidant activity was estimated using the DPPH (2,2′-diphenyl-1-picrylhydrazyl) radical, following the procedure described by Brand-Williams et al. [27]. Results were expressed as IC_50_ (mg mL^−1^ extract concentration able to inhibit 50% of DPPH). All standard and reagents were purchased from Merc (Darmstadt, Germany). 

### 2.6. Soil Analyses

Before sowing and at harvest (development stage 49), five soil samples for each experimental unit were randomly collected at 0–10 cm depth. The five samples were thoroughly mixed to obtain a global soil sample, which was sieved (<2 mm) to remove plant roots, fauna, and debris before analysis. Soil chemical analyses were carried out according to the methods described in “DM 13/09/1999 GU N°248 21/10/1999”. Genomic DNA was extracted from three global soil sample replications utilizing the NucleoSpin^®^ Soil kit (Macherey Nagel, Germany), following the manufacturer’s protocol. DNA quantification of the samples was performed using the Qubit dsDNA HS (High Sensitivity) Assay Kit and a Qubit Fluorometer (ThermoFisher Scientific, Waltham, MA, USA). Replications of each sample were pooled in equimolar mixtures, and samples were sent for 16S rRNA sequencing to Bio-Fab Research Srl (Rome, Italy) as previously described [28].

### 2.7. Data Handling and Statistical Analysis

With respect to the results of the field experiments, a *t*-test was applied to test the differences (α = 0.001) between Control and treated samples by cultivar (XLSTAT 2016—Addinsoft, Paris, France).

16S rRNA sequencing classifications were obtained utilizing the SILVA 132 database (https://www.arb-silva.de/, accessed on 27 February 2021) and the amplicon sequence variants (ASVs) method. Results were processed by QIIME2 [29], and diversity indices were calculated using the R (R Foundation for Statistical Computing, Vienna, Austria) statistical package vegan v. 2.5-7 [30].

## 3. Results

**Figure 1:**
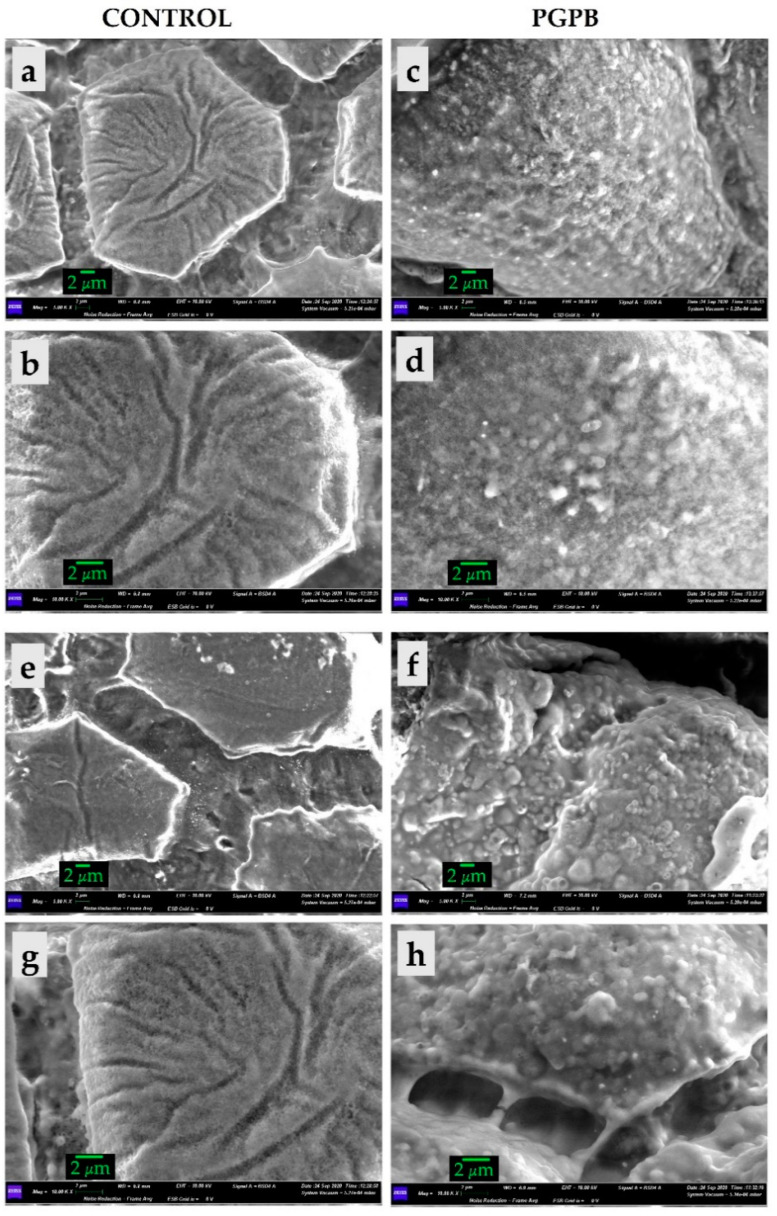
Scanning electron microscope (SEM) micrographs obtained for (**a**) Control (CNT) and inoculated (PGPB) *Allium cepa* seeds the same day of the inoculation (**a**–**d**) and after 30 days (**e**–**g**). Magnitudes: 5000× (**a**,**c**,**e**,**g**) and 10,000× (**b**,**d**,**f**,**h**). Scale bars 2 µm.

To evaluate the bacterial adhesion on seed surfaces, SEM observations of Control and inoculated seeds were carried out. Control seeds showed clear surfaces, while high bacterial adhesion was observed under the PGPB condition (Figure 1A). The bacteria were able to adhere to the seed surfaces and establish aggregation structures that allowed them to persist for up to 30 days (Figure 1B). We also tested inoculum persistence on seeds kept in the dark at room temperature. After 30 days, the number of bacteria present on the seeds remained unchanged. After 60 days, the number of bacteria decreased by 0.5 Log CFU g^−1^. 

**Figure 2:**
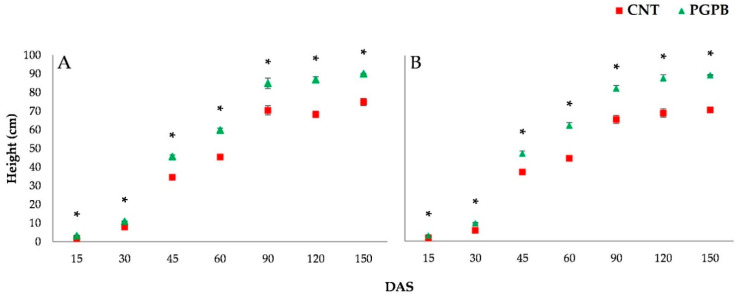
Height dynamics recorded for Meranto (**A**) and Moondance (**B**). In the figure: CNT = Control; PGPB = treated seed; DAS = days after sowing; * = *p* < 0.001.

**Figure 3:**
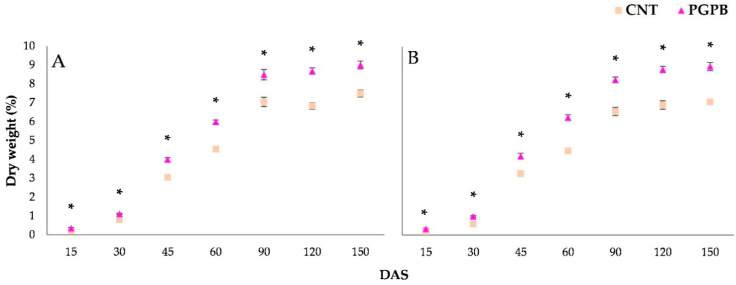
Dry weight dynamics recorded for Meranto (**A**) and Moondance (**B**). In the figure: CNT = Control; PGPB = treated seed; DAS = days after sowing; * = *p* < 0.001.

**Figure 4:**
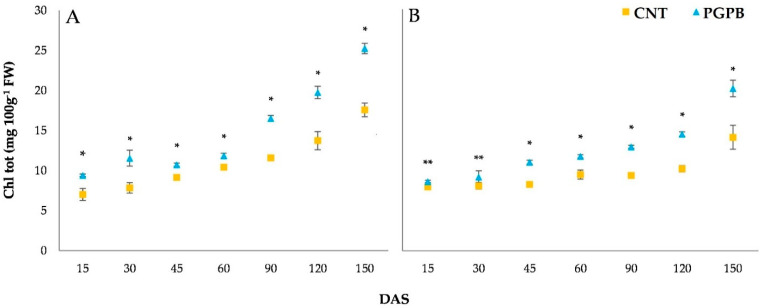
Dynamics of the total chlorophyll content (mg 100g^−1^ FW) recorded for Meranto (**A**) and Moondance (**B**). In the figure: CNT = Control; PGPB = treated seed; DAS = days after sowing; * = *p* < 0.001; ** = *p* < 0.01.

During growth, heights, dry weight, and chlorophylls of the aerial parts were monitored. Figure 2 shows the recorded height dynamics for Meranto and Moondance crops from 15 to 150 DAS. The height of the aerial part was significantly affected (*p* < 0.05) by the bacterial treatment. Throughout the entire growth period, the PGPB condition recorded higher values than the Control, reaching a final average increase of 18% at 150 DAS. Similar trends were also shown by dry weight dynamics (Figure 3); the PGPB condition recorded a final average increase of 23% over the Control. The improvement induced by the bacterial treatment of the seeds was also emphasized by the chlorophyll content. As shown in Figure 4, the treated plants (PGPB) recorded significant increases in chlorophyll content compared to the Control until late maturation (150 DAS), at which an average increase of 43% was recorded. 

**Figure 5:**
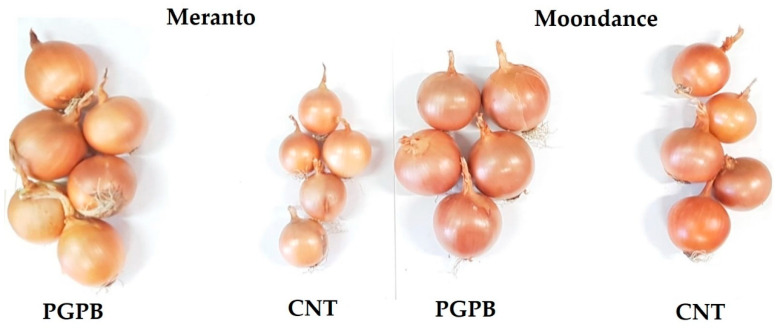
Bulbs harvested from the Control (CNT) and treated (PGPB) experimental units of Meranto and Moondance crops.

At harvest, the total yields (t ha^−1^) were estimated. For the Moondance cultivar, the PGPB condition recorded an average increase of 14%, while for Meranto, the average was 12%. These increases were related to the greater biomass developed by the bulbs in response to bacterial inoculation, which is also easily understandable from Figure 5. 

For both Meranto and Moondance varieties, greater dry weights of bulbs were recorded (*p* < 0.001), with an average increase of 3% (Table 1). 

The bulbs were also analyzed for total phenolic contents and antioxidant activity (Table 1). The bacterial treatment was able to induce an increase in phenols in both Meranto and Moondance bulbs (*p* < 0.05), stimulating the production and accumulation of secondary metabolites. The presence of a larger amount of phenols and possibly other bioactive molecules also induced an increase in antioxidant activity (*p* < 0.01).

To evaluate the inoculum effects on soil fertility status, pre-sowing and post-harvest soil samples were subjected to physico–chemical characterization and DNA extraction and 16S rRNA analysis. Soil physico–chemical changes recorded for Meranto and Moondance experimental conditions were similar (Table 2). Regarding the pre-sowing soil, PGPB samples recorded increases in total organic C and organic matter. Conversely, the Controls recorded decreases in the same parameters. The presence of the inoculum also induced a decrease in Na^+^ and electrical conductivity and a change in the composition of soil nutrients. Compared to the soil collected at pre-sowing, the contents of Ca^++^, Mg^++^, and K^+^ decreased in the Control samples but not in those inoculated with PGPB, while for the available phosphorus, there was an increase in the PGPB samples and a decrease in those of the Control.

16S rRNA gene analysis allowed us to obtain the characterization of the soil microbial community of archaea and bacteria. At the ecological level, compared to the Control soil, the microbial community was positively influenced in terms of diversity and richness. The number of individuals and the Shannon-Wiener H’ and Chao-1 diversity indexes were higher than those of the Control (Table 3).

**Figure 6:**
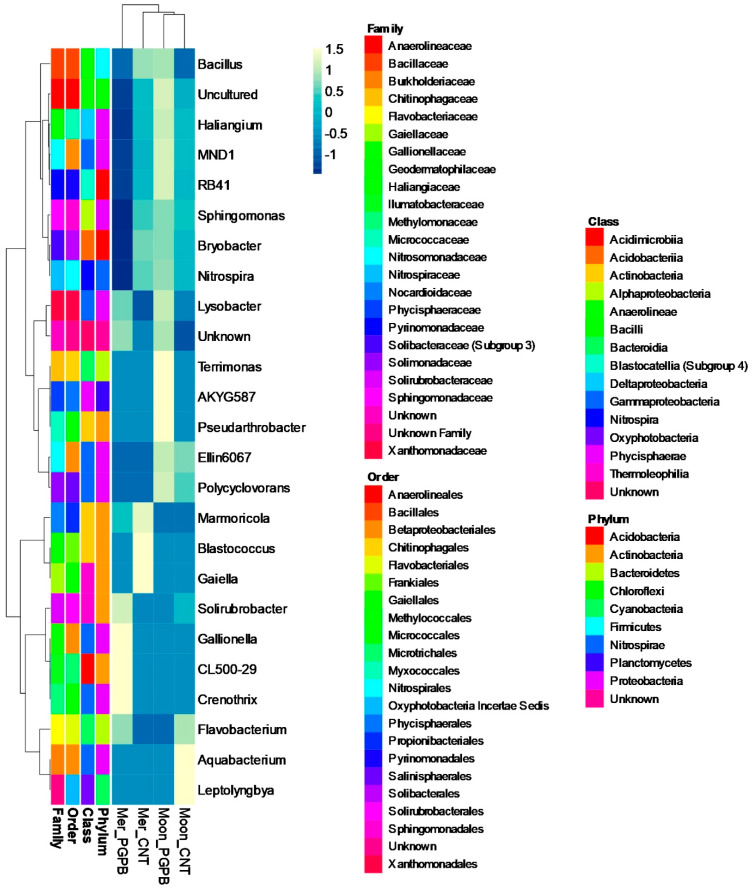
Heat-map and clustering of the soil microbial community composition of the Control (CNT) and treated (PGPB) experimental conditions of Meranto (Mer) and Moondance (Moon) crops. The color changes in the panel show the percentage occupied by the genus inside each sample (color scale at the top). Taxonomy is indicated by the color keys on the right.

The microbial community recorded changes in terms of composition. As shown in Figure 6, the ASVs (amplicon sequence variants) association at the genus level allowed us to establish how the microbial community was changed by bacterial presence. In general, the PGPB treatment recorded increases in the richness of most genera compared to the Control (including the unknown group), with an increase in the *Lysobacter* genus in both Meranto and Moondance PGPB samples. This indicates a cultivar-dependent community and PGPB-dependent enrichment. The ASVs of all samples were mainly associated with unknown Bacteria (30% on average) and Chloroflexi and Proteobacteria phyla. 

## 4. Discussion

The use of biostimulants in agriculture is steadily increasing around the world due to the effectiveness and sustainability of this fertilization approach. The growing use of these innovative products, together with the growing global food demand and the worsening of environmental problems, requires the formulation of new biostimulants. Nevertheless, although plant biostimulants prove to be a valid alternative to synthetic fertilizers, it is necessary to create a valid scientific background that can help the diffusion of these products and sustainable agriculture. Among plant biostimulants, microbial formulations have been investigated since the late 1970s for their plant growth-promoting characteristics. In the last decades, different indirect and direct plant-promotion mechanisms have been ascribed to numerous PGPB strains and their application on seeds/plants by several approaches tested [31]. In this study, we selected one of the simplest application methods, seed inoculation, demonstrating the attachment and survival of microorganism for up to 30 days. This approach has already been proven to be valid a strategy for the improvement of various open field cultivations, both in research and in pre-commercial trials [32]. The ability of bacteria to produce these aggregation structures, known as biofilms, is a fundamental characteristic generally attributed to strains belonging to PGPB and is a key element for their association with plants [4]. Seed soaking with inoculants allows for a good long-term survival of microorganisms, transporting them directly to the plant’s rhizosphere [32], where they can increase plant growth through numerous mechanisms [33]. Biometric results obtained from onion crops (i.e., height, chlorophylls, and dry matter) and bulbs (i.e., yield, dry weight, total phenolic content, and antioxidant activity) showed that seed bacterial treatment improved plant growth and development. Our results are consistent with many previous studies on different crops which recorded similar inoculation effects on soil fertility status [34,35] and on crops. Among those related to onion crops, seed treatment with inoculation of *Azotobacter chroococcum* MF135558 and adhesive agents, in the presence of a 75% dose of NK, improved the growth and yield of plants in a field experiment [36]. Samayoa et al. [37] reported effective isolation and reinoculation of *Leifsonia* sp., *Bacillus megaterium*, and *Pantoea* sp. on onions in combination with a half-dose of chemical fertiliser, recording an improvement in growth during the vegetative stage under pot cultivation. Tinna et al. [38] reported that the integrated use of an *Azotobacter* sp., *Sphingobacterium* sp., and *Burkholderia* sp. consortium as a root dip of seedlings along with mineral fertilizers resulted in improved onion plant growth, bulb yield, and bulb quality parameters. These positive results are usually linked to the production of a certain amount plant growth-promoting (PGP) substances such as indole-3-acetic acid, which promotes the development of root length, volume, and cell division [39] as well as chlorophyll synthesis [40]. N_2_-fixation is another PGP strategy that improves N uptake by plants [41] and the synthesis of siderophores, enzymes, hormones, and essential elements [42], resulting in an increase in plant dry matter [43]. The improved uptake of nutrients mediated by bacteria affects not only nitrogen but also other macro- and micronutrients. The chemical analyses conducted on the soil showed how seed inoculation promoted the release of available phosphorus and the increase in total organic carbon and organic matter. The increase in available phosphorus, without changing the soil composition, is related to the ability of bacteria (PSB) to mobilize insoluble forms of phosphorus, converting them into forms accessible to the plant, and has been described for many genera (*Rhizobium*, *Pseudomonas*, *Azotobacter,* and *Bacillus*) [44]. Microbial inoculation is one of the tools used to modify the characteristics of the soil in productive landscapes. Maintaining good organic carbon levels can be achieved by carbon-sequestering microbial inoculants with the ability to promote carbon sequestration and soil storage [45]. Several PGPB have been shown to have the ability to increase soil carbon levels. However, organic carbon arises from interactions between roots and the rhizospheric microbial community and is influenced by many environmental factors (e.g., nutrient availability, pH, moisture, climatic conditions, atmospheric CO_2_) [46]. The presence of bacterial inoculum also promotes positive changes in the richness and composition of the soil microbial community. How PGPB induce this modification has not yet been clarified [47]. However, it must always be taken into account that also soil composition and plant cultivar influence microbial community structure [48,49]. The abundance and distribution of microorganisms within a community can be modified by the PGPB application [50]. Therefore, to try to establish how and to what extent PGPB can affect the soil microbial community, a multidisciplinary study with the evaluation of all these variables should be carried out.

## 5. Conclusions

The present study allowed us to estimate the effects of seed inoculation on productive onion landscapes. The bacterial consortium of the four selected strains *Azospirillum brasilense*, *Burkholderia ambifaria*, *Gluconacetobacter diazotrophicus,* and *Herbaspirillum seropedicae* was tested in open field experiments on onion. Evaluation of biometrical parameters on plant and bulbs, as well as soil physico–chemical and molecular characterization, revealed that seed treatment improved plant growth and development, crop yield and quality, and the soil fertility status. These results, together with other previously published and unpublished research, confirm the suitability of this consortium as a biostimulating agent in sustainable agriculture. The optimization of large-scale inoculum production procedures as well as the stability of the formulation should be investigated. Furthermore, future studies should be directed towards the creation of scientific evidence on the suitability of this inoculant application on other plant species and the elucidation of the mechanisms of plant-growth improvement and fertility status and changes in the microbial community. Our results suggest that seed inoculation with this consortium could be used in productive onion landscapes as a biostimulating agent in place of chemical fertilizers.

## Figures and Tables

**Table 1:** Dry weight, total phenolic content, and antioxidant activity of bulbs of Meranto and Moondance cultivars.

Index		Dry Weight (%)	Total Phenolic Content (mg GAE g-1 DW)	Antioxidant Activity (IC50)
Moondance	Control	14.28	4.76	150.92
	PGPB	14.73	5.96	12.71
	*t*-test	*	***	**
Meranto	Control	14.21	3.97	93.32
	PGPB	14.70	4.97	75.10
	*t*-test	*	***	*

*t*-test: * = *p* < 0.001; ** = *p* < 0.01; *** = *p* < 0.05.

**Table 2:** Soil chemical analysis for Meranto and Moondance experimental conditions.

Parameter	Moondance			Meranto		
	Pre-Sowing	Control	PGPB	Pre-Sowing	Control	PGPB
pH	7.6	7.7	7.6	7.7	7.7	7.5
Total N (g Kg^−1^)	2.2	2.1	2.2	2.0	1.9	2.0
Total Organic Carbon (g Kg^−1^)	16.8	15.6	18.5	16.5	16.3	18.0
Organic Matter (g Kg^−1^)	29.0	27.0	32.0	28.4	28.0	31.0
Electric Conductivity (μS cm^−1^)	0.45	0.42	0.32	0.40	0.40	0.33
Na^+^ (mg Kg^−1^)	33.0	38.0	15.0	31.0	28.0	18.5
Ca^++^ (mg Kg^−1^)	3022.0	2895.5	3023.2	3132.5	2998.0	3108.5
Mg^++^ (mg Kg^−1^)	148.5	130.5	153.0	145.0	123.2	126.5
K^+^ (mg Kg^−1^)	489.0	383.0	413.5	164.5	138.0	182.2
Available P (mg P_2_O_5_ Kg^−1^)	323.0	172.0	460.5	282.0	144.2	227.0

**Table 3:** Ecological indexes calculated from Illumina sequencing results.

Index		Individuals	Shannon-Wiener H’	Chao-1
Moondance	Control	7431	6.15	628
	PGPB	9627	6.33	753
Meranto	Control	4046	5.66	363
	PGPB	5209	5.79	429

## References

[1] Rouphael Y., Colla G. (2020). Editorial: Biostimulants in Agriculture. Front. Plant Sci..

[2] du Jardin P. (2015). Plant biostimulants: Definition, concept, main categories and regulation. Sci. Hortic..

[3] Colla G., Nardi S., Cardarelli M., Ertani A., Lucini L., Canaguier R., Rouphael Y. (2015). Protein hydrolysates as biostimulants in horticulture. Sci. Hortic..

[4] Djebaili R., Pellegrini M., Smati M., Del Gallo M., Kitouni M. (2020). Actinomycete Strains Isolated from Saline Soils: Plant-Growth-Promoting Traits and Inoculation Effects on Solanum lycopersicum. Sustain. J. Rec..

[5] Khoshru B., Mitra D., Khoshmanzar E., Myo E.M., Uniyal N., Mahakur B., Das Mohapatra P.K., Panneerselvam P., Boutaj H., Alizadeh M. (2020). Current scenario and future prospects of plant growth-promoting rhizobacteria: An economic valuable resource for the agriculture revival under stressful conditions. J. Plant Nutr..

[6] Mahmood A., Turgay O.C., Farooq M., Hayat R. (2016). Seed biopriming with plant growth promoting rhizobacteria: A review. FEMS Microbiol. Ecol..

[7] Bashan Y., De-Bashan L.E. (2010). How the Plant Growth-Promoting Bacterium Azospirillum Promotes Plant Growth—A Critical Assessment. Adv. Agron..

[8] Raja P., Uma S., Gopal H., Govindarajan K. (2006). Impact of bio inoculants consortium on rice root exudates, biological nitrogen fixation and plant growth. J. Biol. Sci..

[9] Rajasekar S., Elango R. (2011). Effect of microbial consortium on plant growth and improvement of alkaloid content in Withania somnifera ( Ashwagandha). Curr. Bot..

[10] Cappellari L.D.R., Santoro M.V., Nievas F., Giordano W., Banchio E. (2013). Increase of secondary metabolite content in marigold by inoculation with plant growth-promoting rhizobacteria. Appl. Soil Ecol..

[11] Döbereneir J., Balows A., Trüper H.G., Dworkin M., Harder W., Schleifer K.-H. (1991). The genera Azospirillum and Herbaspirillum. The Prokaryotes: A Handbook on the Biology of Bacteria: Ecophysiology, Isolation, Identification, Applications.

[12] Moretti L.G., Crusciol C.A.C., Kuramae E.E., Bossolani J.W., Moreira A., Costa N.R., Alves C.J., Pascoaloto I.M., Rondina A.B.L., Hungria M. (2019). Effects of growth-promoting bacteria on soybean root activity, plant development, and yield. Agron. J..

[13] Sarma B.K., Yadav S.K., Singh S., Singh H.B. (2015). Microbial consortium-mediated plant defense against phytopathogens: Readdressing for enhancing efficacy. Soil Biol. Biochem..

[14] Shahzad S., Khan M.Y., Zahir Z.A., Asghar H.N., Chaudhry U.K. (2017). Comparative Effectiveness of Different Carriers To Im-prove the Efficacy. Pakistan J. Bot..

[15] Dwivedi M., Amaresan N., Senthil Kumar M., Annapurna K., Kumar K., Sankaranarayanan A. (2020). Gluconobacter. Beneficial Microbes in Agro-Ecology.

[16] Monteiro R.A., Balsanelli E., Wassem R., Marin A.M., Brusamarello-Santos L.C.C., Schmidt M.A., Tadra-Sfeir M.Z., Pankievicz V.C.S., Cruz L.M., Chubatsu L.S. (2012). Herbaspirillum-plant interactions: Microscopical, histological and molecular aspects. Plant Soil.

[17] Castanheira N., Dourado A., Kruz S., Alves P., Delgado-Rodríguez A., Pais I.P., Semedo J.N., ScottiCampos P., Sanchez C.R.M., Borges N. (2016). Plant growth-promoting Burkholderia species isolated from annual ryegrass in Portuguese soils. J. Appl. Microbiol..

[18] Fiore A., Laevens S., Bevivino A., Dalmastri C., Tabacchioni S., Vandamme P., Chiarini L. (2001). Burkholderia cepacia complex: Distribution of genomovars among isolates from the maize rhizosphere in Italy. Environ. Microbiol..

[19] Kloepper J.W., Leong J., Teintze M., Schroth M.N. (1980). Enhanced plant growth by siderophores produced by plant growth-promoting rhizobacteria. Nat. Cell Biol..

[20] Thomloudi E.-E., Tsalgatidou P., Douka D., Spantidos T.-N., Dimou M., Venieraki A., Katinakis P. (2019). Multistrain versus single-strain plant growth promoting microbial inoculants—The compatibility issue. Hell. Plant Prot. J..

[21] Botta A.L., Santacecilia A., Ercole C., Cacchio P., Del Gallo M. (2013). In vitro and in vivo inoculation of four endophytic bacteria on Lycopersicon esculentum. New Biotechnol..

[22] Pagnani G., Pellegrini M., Galieni A., D’Egidio S., Matteucci F., Ricci A., Stagnari F., Sergi M., Sterzo C.L., Pisante M. (2018). Plant growth-promoting rhizobacteria (PGPR) in Cannabis sativa ‘Finola’ cultivation: An alternative fertilization strategy to improve plant growth and quality characteristics. Ind. Crop. Prod..

[23] Pace L., Pacioni G., Spanò L. (2004). In vitro propagation of *Artemisia petrosa* ssp. eriantha: Potential for the preservation of an endangered species. Plant Biosyst..

[24] Pagnani G., Galieni A., Stagnari F., Pellegrini M., Del Gallo M., Pisante M. (2020). Open field inoculation with PGPR as a strategy to manage fertilization of ancient Triticum genotypes. Biol. Fertil. Soils.

[25] Raikhel N.V. (2003). Plant Physiology’s Best Paper Award 2002. Plant Physiol..

[26] Singleton V.L., Rossi J.A. (1965). Colorimetry of Total Phenolics with Phosphomolybdic-Phosphotungstic Acid Reagents. Am. J. Enol. Vitic..

[27] Brand-Williams W., Cuvelier M., Berset C. (1995). Use of a free radical method to evaluate antioxidant activity. LWT.

[28] Vaccarelli I., Matteucci F., Pellegrini M., Bellatreccia F., Del Gallo M. (2021). Exploring Microbial Biosignatures in Mn-Deposits of Deep Biosphere: A Preliminary Cross-Disciplinary Approach to Investigate Geomicrobiological Interactions in a Cave in Central Italy. Front. Earth Sci..

[29] Bolyen E., Rideout J.R., Dillon M.R., Bokulich N.A., Abnet C.C., Al-Ghalith G.A., Alexander H., Alm E.J., Arumugam M., Asnicar F. (2019). Reproducible, interactive, scalable and extensible microbiome data science using QIIME 2. Nat. Biotechnol..

[30] Oksanen J., Blanchet F., Friendly M., Kindt R., Legendre P., McGlinn D., Minchin P., O’Hara B., Simpson G., Solymos P. The Vegan Package. https://cran.r-project.org/web/packages/vegan/vegan.pdf.

[31] Bashan Y., De-Bashan L.E., Prabhu S.R., Hernandez J.-P. (2014). Advances in plant growth-promoting bacterial inoculant technology: Formulations and practical perspectives (1998–2013). Plant Soil.

[32] O’Callaghan M. (2016). Microbial inoculation of seed for improved crop performance: Issues and opportunities. Appl. Microbiol. Biotechnol..

[33] Babalola O.O. (2010). Beneficial bacteria of agricultural importance. Biotechnol. Lett..

[34] Orhan E., Esitken A., Ercisli S., Turan M., Sahin F. (2006). Effects of plant growth promoting rhizobacteria (PGPR) on yield, growth and nutrient contents in organically growing raspberry. Sci. Hortic..

[35] Ullah A., Bano A. (2019). Role of PGPR in the reclamation and revegetation of saline land. Pak. J. Bot..

[36] Afify A., Hauka F., ElSawah A. (2019). Plant Growth-Promoting Rhizobacteria enhance Onion (*Allium cepa* L.) productivity and minimize requisite chemical fertilization. Environ. Biodivers. Soil Secur..

[37] Samayoa B.E., Shen F.-T., Lai W.-A., Chen W.-C. (2020). Screening and Assessment of Potential Plant Growth-promoting Bacteria Associated with *Allium cepa* Linn.. Microbes Environ..

[38] Tinna D., Garg N., Sharma S., Pandove G., Chawla N. (2020). Utilization of plant growth promoting rhizobacteria as root dipping of seedlings for improving bulb yield and curtailing mineral fertilizer use in onion under field conditions. Sci. Hortic..

[39] Wahyudi A.T., Priyanto J.A., Afrista R., Kurniati D., Astuti R.I., Akhdiya A. (2019). Plant Growth Promoting Activity of Actinomycetes Isolated from Soybean Rhizosphere. Online J. Biol. Sci..

[40] Czerpak R., Dobrzyń P., Krotke A., Kicińska E. (2002). The Effect of auxins and salicylic acid on chlorophyll and carotenoid contents in Wolffia arrhiza (L.) Wimm. (Lemnaceae) growing on media of various trophicities. Polish J. Environ. Stud..

[41] Boddey R.M., Urquiaga S., Alves B.J., Reis V. (2003). Endophytic nitrogen fixation in sugarcane: Present knowledge and future applications. Plant Soil.

[42] Badawi F., Biomy A., Desoky A. (2011). Peanut plant growth and yield as influenced by co-inoculation with Bradyrhizobium and some rhizo-microorganisms under sandy loam soil conditions. Ann. Agric. Sci..

[43] Venieraki A., Dimou M., Pergalis P., Kefalogianni I., Chatzipavlidis I., Katinakis P. (2010). The Genetic Diversity of Culturable Nitrogen-Fixing Bacteria in the Rhizosphere of Wheat. Microb. Ecol..

[44] Kalayu G. (2019). Phosphate Solubilizing Microorganisms: Promising Approach as Biofertilizers. Int. J. Agron..

[45] Ahmed A.A.Q., Odelade K.A., Babalola O.O. (2019). Microbial Inoculants for Improving Carbon Sequestration in Agroecosystems to Mitigate Climate Change. Handbook of Climate Change Resilience.

[46] Song W., Tong X., Liu Y., Li W. (2020). Microbial Community, Newly Sequestered Soil Organic Carbon, and δ15N Variations Driven by Tree Roots. Front. Microbiol..

[47] Ren H., Huang B., Fernández-García V., Miesel J., Yan L., Lv C. (2020). Biochar and Rhizobacteria Amendments Improve Several Soil Properties and Bacterial Diversity. Microorganisms.

[48] Jiang Y., Li S., Li R., Zhang J., Liu Y., Lv L., Zhu H., Wu W., Li W. (2017). Plant cultivars imprint the rhizosphere bacterial community composition and association networks. Soil Biol. Biochem..

[49] Inceoğlu Ö., Salles J.F., Van Elsas J.D. (2012). Soil and Cultivar Type Shape the Bacterial Community in the Potato Rhizosphere. Microb. Ecol..

[50] Hou J., Liu W., Wang B., Wang Q., Luo Y., Franks A.E. (2015). PGPR enhanced phytoremediation of petroleum contaminated soil and rhizosphere microbial community response. Chemosphere.

